# *Syringa vulgaris* leaves powder a novel low-cost adsorbent for methylene blue removal: isotherms, kinetics, thermodynamic and optimization by Taguchi method

**DOI:** 10.1038/s41598-020-74819-x

**Published:** 2020-10-19

**Authors:** Giannin Mosoarca, Cosmin Vancea, Simona Popa, Marius Gheju, Sorina Boran

**Affiliations:** Faculty of Industrial Chemistry and Environmental Engineering, Politehnica University Timisoara, Bd. V. Parvan No. 6, 300223 Timisoara, Romania

**Keywords:** Environmental chemistry, Pollution remediation

## Abstract

In this study, the potential of a new low-cost adsorbent, *Syringa vulgaris* leaves powder, for methylene blue adsorption from aqueous solution was investigated. The adsorbent surface was examined using SEM and FTIR techniques. The experiments were conducted, in batch system, to find out the effect of pH, contact time, adsorbent dose, initial dye concentration, temperature and ionic strength on dye adsorption. The process is best described by Langmuir isotherm and the pseudo second order kinetic model. Maximum adsorption capacity, 188.2 (mg g^−1^), is better than other similar adsorbent materials. Thermodynamic parameters revealed a spontaneous and endothermic process, suggesting a physisorption mechanism. A Taguchi orthogonal array (L_27_) experimental design was used to determine the optimum conditions for the removal of dye. Various desorbing agents were used to investigate the regeneration possibility of used adsorbent. Results suggest that the adsorbent material is very effective for removal of methylene blue from aqueous solutions.

## Introduction

Dyes are extensively used in numerous industries like textile, plastic, dye, leather, paper, rubber, cosmetics, food, carpet and printing^1–5^. The wastewaters resulted from these industries contain residual dyes which are not bio-degradable and cause serious environmental problems, in general, and water pollution, in particular: visible pollution and damage on the aesthetic nature of effluent water, reduction in the sunlight penetration which affect photosynthesis and biota growth, ecotoxicity risk and bioaccumulation potential danger^1–3,5–7^.

Methylene blue (MB) has wide utilizations in industry and is also used in several important medical applications. It can have some negative effects on human health such as respiratory problem, eye irritation, nausea, vomiting, diarrhea, dyspnea, tachycardia, cyanosis, jaundice and methemoglobinemia^8–12^.

Many conventional methods have been developed to remove dyes from wastewater: coagulation, adsorption, membrane processes, ion exchange, precipitation, chemical oxidation, electrochemical processes, ozonation, zero valent iron reduction, photocatalytic processes and biodegradation. Adsorption process is preferred due to its high efficiency (even for the diluted solutions), low cost, flexibility, ease of operation, design simplicity and availability of adsorbents^2,4,6,10–17^.

Activated carbon is the most used widely adsorbent, but due to its high cost and regeneration difficulty many studies focus on the discovery and development of novel low cost adsorbents like: fly ash, metal hydroxide sludge, red mud, zeolites, siliceous materials, chitosan, bone char, sawdust, sugarcane bagasse, coffee beans, wheat straw, wheat husk, nutshells, longan shell, rice husk, rice bran, corn cobs, cereal chaff, pine cone, coconut coir, shaddock peel, garlic peel, broad bean peels, jack fruit peel, yellow passion fruit peel, durian peel, pomelo peel, pineapple stem, pineapple leaves, spent tea leaves, phoenix tree's leaves, *Salix babilonica* leaves, *Platanus orientalis* leaves, datepalm leaves, guava leaves, lotus leaves, *Hibiscus cannabinus* fiber, roots of *Eichhornia crassipes*, papaya seeds^2,4,8–11,15,16,18–25^.

Various compounds such as cellulose, hemi-cellulose, pectins, lignin, polyphenolics, plant pigments and protein from the tree leaves structure have various functional groups (carboxyl, carbonyl, hydroxyl, amino, nitro) which can provide active sites for dye binding and make adsorption process possible^2,20,23,26^.

*Syringa vulgaris* L. (lilac or common lilac) is a large shrub or small tree native to the Balkan Peninsula, which growing to 6–7 m tall. It has been naturalized and cultivated, on a large scale, as an ornamental plant in Europe and North America. Is also found in the wild in widely scattered sites, usually in the human habitations vicinity. The lilac is a very popular ornamental plant in gardens and parks, because of its flowers attractiveness, having a relatively varied range of colors, and an extraordinary scent^27–29^.

In this study, the *Syringa vulgaris* leaves have been used as a new low-cost adsorbent for methylene blue removal from aqueous solution. The effects of main parameters (pH, contact time, adsorbent dose, initial dye concentration, temperature and ionic strength) that may influence the adsorption process were investigated. Equilibrium and kinetic modeling, thermodynamic study, optimization using Taguchi approach and the regeneration possibility of used adsorbent were also conducted.

## Materials and methods

Mature *Syringa vulgaris* leaves (SVL) were collected from a lilac tree from Buzias, Timis County, Romania, washed with distilled water, dried at room temperature for 3 days and afterwards in an air oven at 90 °C for 24 h. The dried mass was grounded with a mechanical grinder, passed through a 2 mm sieve and washed with distilled water to remove the turbidity and color. Finally, the washed material was dried for 5 h, at 105 °C, in an air oven.

A scanning electron microscope Quanta FEG 250 (at 3000 × magnitude) and Shimadzu Prestige-21 FTIR spectrophotometer were used to characterize the adsorbent, before and after adsorption. The determination of point of zero charge (PZC) was realized through the solid addition method^3^. According to this method, samples of 50 mL of KNO_3_ solution with a concentration of 0.1 (mol L^−1^) are used. The pH of each sample was adjusted from 2 to 12 using 1 M HNO_3_ or 1 M NaOH. 1 g of adsorbent material was added to each sample. The samples were shaken for 5 h and then left at rest for 24 h. The final pH was measured and the difference between the initial and final pH values (ΔpH = pH_i_ − pH_f_) was plotted against the pH. The point of intersection between the resulting curve with pH axis represents the value corresponding to the point of zero charge.

Merck analytical grade chemicals were used in the experiments. The studies were conducted in Erlenmeyer flasks (150 mL) at constant mixing intensity. For mixing the adsorbent with the dye solution was used an M.T.A. 609/A shaker. The pH adjustment was performed with 0.1 M NaOH and HCl solutions. The ionic strength effect was tested using NaCl as background electrolyte. The methylene blue concentration was measured by a UV–VIS spectrophotometer Specord 200 PLUS at 664 nm wavelength. For each experiment, there were three independent replicates.

Non-liniar and linear forms of Langmuir and Freundlich isotherms and pseudo-first order and pseudo-second order kinetic models were performed according to methods described in literature^5,9,11,12,16,19,20,24,25,30,31^. The values of determination coefficient (R^2^), sum of square error (SSE), chi-square (χ^2^) and average relative error (ARE) were taking into consideration to establish the best-fitting model for the adsorption process^30^. The thermodynamic parameters (standard Gibbs free energy change, standard enthalpy change and standard entropy change) were computed by using data of MB adsorption at temperatures of 285, 296 and 306 K using the equations described elsewhere^21,25,32–34^.

In order to obtain the optimum conditions for methylene blue adsorption, Taguchi (L_27_) experimental design was used. The effect of six controllable factors (pH, contact time, adsorbent dose, initial dye concentration, temperature and solution ionic strength) at three levels on the methylene blue removal efficiency was investigated (see Supplementary Information, Table S1). The Taguchi method uses an orthogonal array (OA) for experimental design and analyzes the signal to noise ratio (S/N) to assess the experimental results. The larger-the-better S/N ratio option was determined because the highest adsorption efficiency was taken into consideration^35–38^. An analysis of variance (ANOVA) was used to assess the Taguchi model results and to determine the percent contribution of each factor on the dye removal efficiency^35,37–39^. Minitab 19 software was utilized to perform the required calculations.

For the desorption experiments the dye-loaded adsorbent was agitated, at constant mixing intensity, with different desorbing solutions (0.1 M HCl, 0.1 M NaOH, distilled water) and equilibrated for 2 h.

## Results and discussion

### Adsorbent characterization

**Figure 1:**
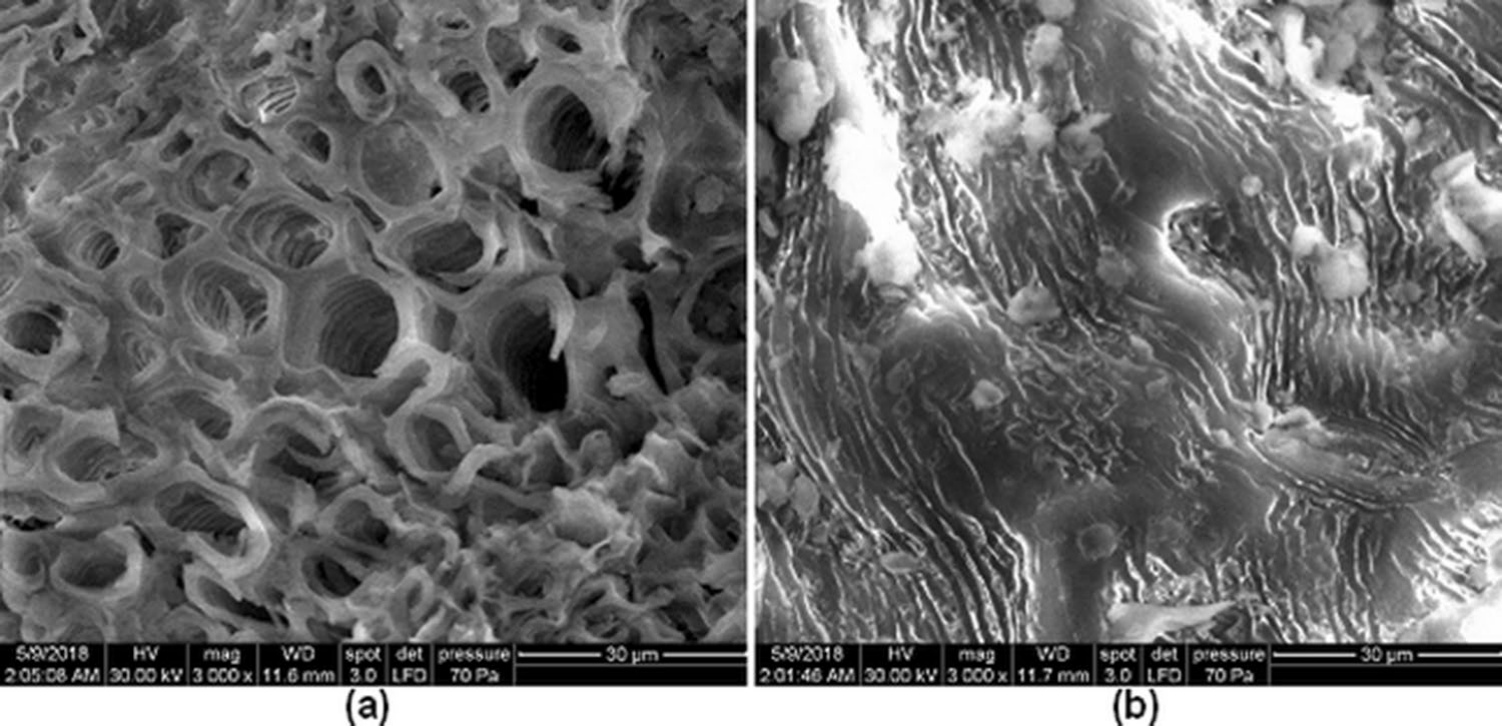
SEM images of adsorbent material: (**a**) before and (**b**) after dye adsorption.

The surface morphologies of the adsorbent before and after adsorption are shown in Fig. 1. Before adsorption (Fig. 1a) the surface has many pores with different shape and sizes which provide a large number of active sites available for dye adsorption. After adsorption (Fig. 1b) MB molecules fill these pores and the surface of adsorbent becomes saturated, covered by dye molecule.

The FTIR analysis suggests that the main ingredients of adsorbent material are cellulose, hemicellulose and lignin. The FTIR spectra of adsorbent material before and after methylene blue adsorption, presented in Supplementary Information (Figure S1), show following different specific peaks for main functional group: 3382 cm^−1^—OH stretching vibration of phenols, carboxylic acids and alcohols as in lignin, pectin and cellulose^15^, 2933 cm^−1^—CH stretching of CH_2_^40^, 1647 cm^−1^—C=O stretching characteristic of lignin or hemicellulose^41,42^, 1422 cm^−1^—C–H deformation in lignin^43,44^, 1255 cm^−1^—C–O stretching and CH or OH bending indicate the existence of hemicellulose structures^25,45,46^, 1026 cm^−1^—C–O, C–O–H, C–O–C, C–C, ring stretching vibration in cellulose and hemicellulose^47^, 609 cm^−1^—the bending modes of aromatic compounds of cellulose^48,49^. The differences between the wavenumber of the peaks before and after adsorption are small (less than 10 cm^−1^) which indicate that the methylene blue adsorption mechanism could include an ion-exchange mechanism or physical interaction^50^.

The point of zero charge (PZC) is a parameter that indicates the adsorption ability on the adsorbent surface. At pHpzc, the net charge of the adsorbent surface is zero. At pH < pHpzc adsorbent surface becomes positively charged and at pH > pHpzc becomes negatively charged. Adsorption of the cationic dye is favored by a negatively charge surface of the adsorbent^2,9,11^. The PZC of adsorbent was determined as 5.77 (see Supplementary Information, Figure S2) and a pH above this value is electrostatically favorable for the methylene blue adsorption process.

### Effect of pH on methylene blue adsorption

**Figure 2:**
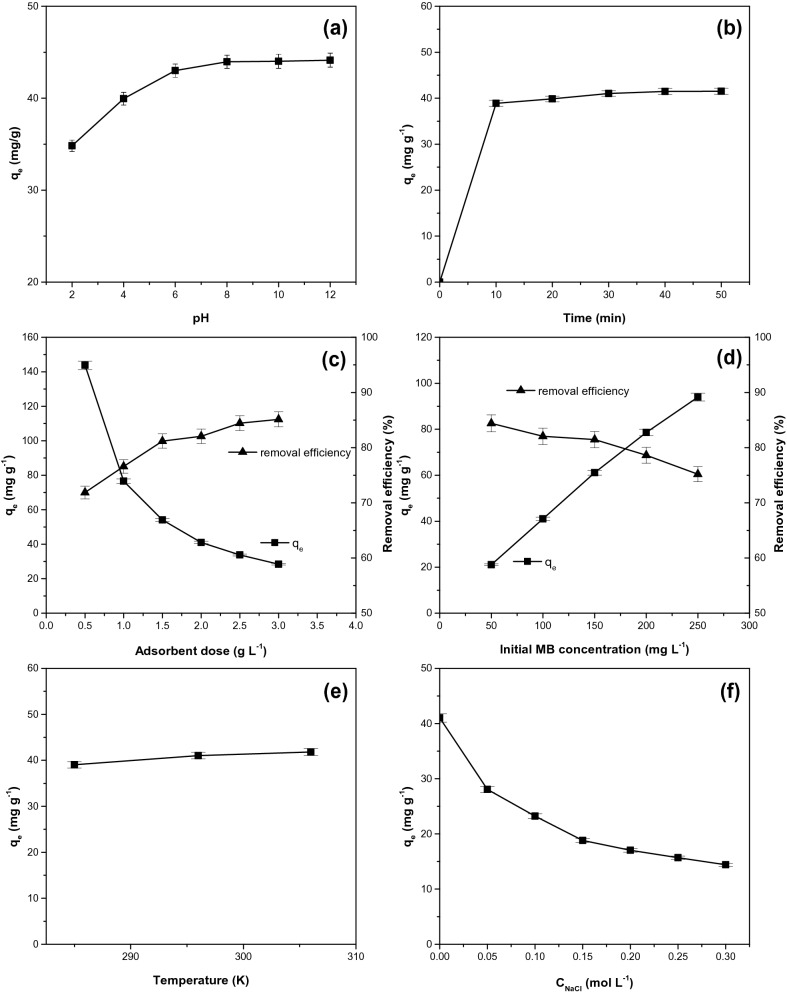
Effect of (**a**) pH, (**b**) time, (**c**) adsorbent dose, (**d**) initial dye concentration, (**e**) temperature, (**f**) ionic strength on methylene blue adsorption onto the adsorbent efficiency. (Adsorption conditions: (**a**): initial methylene blue concentration: 100 mg L^−1^; contact time: 30 min; adsorbent dosage: 2 g L^−1^; temperature: 296 K, (**b**): pH 7, others identical to Fig. 3a, (**c**): contact time: 30 min, others identical to Fig. 3b, (**d**): adsorbent dosage: 2 g L^−1^, others identical to Fig. 3c, (**e**): initial dye concentration: 100 mg L^−1^, others identical to Fig. 3d, (**f**): temperature: 296 K, others identical to Fig. 3d).

Figure 2a showed the adsorption capacity of dye onto *Syringa vulgaris* leaf at different pH. With pH increasing from 2 to 12, the adsorption capacity increases from 34.8 (mg g^−1^) to 44.1 (mg g^−1^). In the pH range 2–6 the increase is more pronounced.

Similar trends were observed for the adsorption of methylene blue onto phoenix tree's leaves^20^, banana leaves^58^. Below pHpzc = 5.77 the adsorbent surface is positively charged and an electrostatic repulsion with the cationic dye appears, which prevent the adsorption. With the increase of the pH, the surface of adsorbent became negatively charged which will facilitate the electrostatic attraction with the cationic dye molecules and the adsorption capacity will increase^11,15,21,22^. Adsorption capacity does not increase greatly with pH, on the range 6–12 being practically constant, which indicated that the electrostatic interaction was not the only mechanism for dye adsorption^21,22^.

### Effect of contact time on methylene blue adsorption

According to Fig. 2b, a rapid adsorption of dye occurs in the first 10 min and, thereafter, the increase of adsorption capacity became much slower. The equilibrium was reached after 30 min. Rapid increase of the adsorption capacity at the beginning of the process is due to the availability of a large exposed surface active sites number for methylene blue adsorption. The available active sites are gradually filled up with the increasing of contact time, leading to the slow increase of adsorption capacity. At this stage it can be assumed that dye diffusion occurs in pores of the adsorbent^2,21^. It is possible to form a dye monolayer on the adsorbent surface, which is indicated by the decrease of the low adsorption rate^24^. The equilibrium times reported for different adsorbent were: 30 min for carrot stem powder^3^, carrot leaves powder^3^ and *Arthrospira platensis* biomass^7^, 60 min for marine macro-alga of *Euchema Spinosum*^9^, CO_2_ activated corn cob carbon^10^ and pineapple leaf powder^25^, 70 min for *Platanus orientalis* leaf powder^23^, 80 min for maize silk powder^15^, 90 min for steam treated corn cob carbon^10^, 150 min for phoenix tree's leaves^20^ and lotus leaf^21^, 160 min for waste of seeds of *Aleurites Moluccana*^5^ and 200 min for peanut husk^24^.

### Effect of adsorbent dose on methylene blue adsorption

Figure 2c shows the removal efficiency and adsorption capacity at the various dosage of adsorbent material. The adsorption removal efficiency increases with the adsorbent dose due to the increase of adsorbent surface area and availability of a larger number of adsorption active sites^11,20,21,51^. The adsorption capacity decrease from 143.6 to 28.3 (mg g^−1^) when the adsorbent dosage increases from 0.5 to 3.0 (g L^−1^). The decrease of adsorption capacity could be attributed to unsaturation of adsorption sites during the process, whereas, the sites number available for adsorption increases or to aggregation or agglomeration of adsorbent particle which leads to the decrease of total adsorbent surface area and an increase of the diffusion path length^11,20,51^. As with other adsorbent materials (*Salix babylonica* leaves powder^2^, citrus limetta peel^11^, phoenix tree's leaves^20^, lotus leaf^21^, orange peel powder^52^) used for the methylene blue removal from water, a similar effect of the adsorbent dose on the dye removal efficiency and on the adsorption capacity was recorded.

### Effect of initial dye concentration on methylene blue adsorption

Initial dye concentration has a significant effect on the adsorption process (Fig. 2d). The adsorption capacity increase from 21.1 to 94.0 (mg g^−1^) when methylene blue initial concentration rises from 50 to 250 (mg L^−1^). This could be attributed to the increase of the driving force necessary to overcome the resistance to mass transfer of dye between the aqueous phase and solid phase^2,4,20–23,51^. In addition, increasing the initial dye concentration may favor the number of collisions between the dye cations and the adsorbent material, improving (intensifying) the adsorption process^7^. At the same time, the removal percentage of the dye has decreased from about 84.4 to 75.2%, with an increase in the initial dye concentration. This behavior can be explained by the saturation of the adsorption sites caused by the dye molecules accumulation on the surface of the adsorbent particle^52,53^. These observations regarding the influence of the initial dye concentration onto the adsorption capacity and the removal efficiency are in agreement with the results obtained with other adsorbent materials, such as *Salix babylonica* leaves powder^2^, acid treated kenaf fibre char^4^, eucalyptus barks bio-char^53^.

### Effect of temperature on methylene blue adsorption

The influence of temperature on the adsorption capacity is depicted in Fig. 2e. The adsorption capacity rise from 39.0 to 41.8 (mg g^−1^) when the temperature increased from 285 to 306 K, indicating that the adsorption was endothermic in nature^4,8^. The increase of temperature favors the mobility of the large dye cations and reduces the viscosity of the solution. This fact increases dye molecules diffusion rate in the external boundary layer and internal pores of the adsorbent^4,22^. The positive effect of temperature on the adsorption capacity was reported for acid treated kenaf fibre char^4^, graphene^8^, phoenix tree's leaves^20^, lotus leaf^21^, *Platanus orientalis* leaf powder^23^ and eucalyptus barks bio-char^53^.

### Effect of ionic strength on methylene blue adsorption

Usually, dyeing wastewater may contains high salt concentration and various metal ions leading to high ionic strength and affecting the adsorption process^3,20^. Figure 2f shows the effect of NaCl presence on dye removal from water. The increase of salt concentration from 0 to 0.3 (mol L^−1^) causes a decrease of adsorption capacity from 41.0 to 14.4 (mg g^−1^). This trend could be attributed to the competitive effect between dye cations and Na^+^ ions for the adsorption available sites. When the ionic strength increases, the activity (effective concentration) of methylene blue and the active sites number decrease, so the adsorption capacity is reduced^3,20,21,25^. Similar phenomenon was also observed for adsorption of methylene blue by carrot stem and carrot leaves powders^3^, *Arthrospira platensis* biomass^7^, phoenix tree's leaves^20^, lotus leaf^21^ and pineapple leaf powder^25^.

### Equilibrium and kinetic modeling

Adsorption isotherms are very important, providing information about adsorption mechanism, surface properties and adsorbent capacity under the system condition. Langmuir isotherm assumes that the adsorption takes place by monolayer sorption, without interaction between the adsorbed molecules, on a homogeneous surface. Freundlich isotherm assumes multilayer adsorption on the solid adsorbent heterogeneous surface^48,54,55^.

Figures S3–S5 from Supplementary Information illustrate the Langmuir and Freundlich adsorption isotherms (non-linear and linear forms) for methylene blue adsorption on *Syringa vulgaris* leaves powder. The criterion for their applicability was the greater value for determination coefficient (R^2^) and the smaller values for sum of square error (SSE), chi-square (χ^2^) and average relative error (ARE). Isotherms constants and the error functions values are summarized in Table 1. Langmuir isotherm was found to be best-fitting model for describing the adsorption process, indicating a monolayer adsorption process on a homogenous surface of adsorbent. The value of maximum adsorption capacity 188.2 (mg g^−1^) is better than other similar adsorbents: Neem leaf powder 19.6 (mg g^−1^)^56^, *Salix babylonica* leaves 60.9 (mg g^−1^)^2^, Phoenix tree leaves 80.9 (mg g^−1^)^20^, oil palm leaves 103.0 (mg g^−1^)^57^, banana leaves 109.9 (mg g^−1^)^58^, *Platanus orientalis* leaf 114.9 (mg g^−1^)^23^. The R_L_ value for the methylene blue adsorption was 0.382 suggesting a favorable adsorption.

**Table 1:** Adsorption isotherms and kinetic models constants and the corresponding error functions.

Isotherm model	Parameters	Value	Kinetic model	Parameters	Value
Langmuir non-linear	K_L_ (L mg^−1^)	0.016 ± 0.002	Pseudo-first order non-linear	k_1_ (min^−1^)	0.29 ± 0,03
	q_max_ (mg g^−1^)	186.2 ± 13.3		q_e_,_calc_ (mg g^−1^)	40.84 ± 0.32
	R^2^	0.9972		R^2^	0.9988
	χ^2^	0.18		χ^2^	0.04
	SSE	9.58		SSE	1.25
	ARE (%)	2.40		ARE (%)	17.68
Langmuir linear	K_L_ (L mg^−1^)	0.016 ± 0.003	Pseudo-first order linear	k_1_ (min^−1^)	0.15 ± 0,04
	q_max_ (mg g^−1^)	188.2 ± 7.4		q_e_,_calc_ (mg g^−1^)	27.80 ± 2.11
	R^2^	0.9986		R^2^	0.9693
	χ^2^	6.46 × 10^–5^		χ^2^	0.13
	SSE	1.24 × 10^–6^		SSE	0.24
	ARE (%)	1.03		ARE (%)	31.95
Freundlich non-linear	*K*_f_ (mg g^−1^)	6.30 ± 1.35	Pseudo-second order non-linear	k_2_ (g mg^−1^ min^−1^)	0.026 ± 0.004
	1/n	0.662		q_e_,_calc_ (mg g^−1^)	42.13 ± 0.27
	R^2^	0.9863		R^2^	0.9997
	χ^2^	1.04		χ^2^	0.01
	SSE	48.15		SSE	0.38
	ARE (%)	6.39		ARE (%)	17.13
Freundlich linear	*K*_f_ (mg g^−1^)	4.85 ± 1.05	Pseudo-second order linear	k_2_ (g mg^−1^ min^−1^)	0.022 ± 0.003
	1/n	0.735 ± 0.048		q_e_,_calc_ (mg g^−1^)	42.43 ± 0.35
	R^2^	0.9893		R^2^	0.9998
	χ^2^	1.61 × 10^–3^		χ^2^	1.24 × 10^–4^
	SSE	2.84 × 10^–3^		SSE	5.69 × 10^–5^
	ARE (%)	1.16		ARE (%)	0.59

Adsorption kinetics is very important since it provides information regarding adsorption mechanism, process efficiency and its applicability on an industrial scale^5,57,59^. The pseudo-first order and pseudo-second order kinetic models (non-linear and linear forms) for methylene blue adsorption are presented in Supplementary Information, Figures S6–S8. Kinetic parameters for these models and the error functions values are reported in Table 1 and indicate that pseudo-second order kinetic model best describes the adsorption process. Also, there is good agreement between calculated and experimental value of equilibrium adsorption capacity, thus confirming that this kinetic model is more suitable to describe the dye adsorption on the adsorbent material. Scientific literature reported that the methylene blue adsorption is described by pseudo-second-order kinetic model for *Salix babylonica* leaves^2^, waste of seeds of *Aleurites Moluccana*^5^, *Arthrospira platensis* biomass^7^, marine macro-alga of *Euchema Spinosum*^9^, citrus limetta peel waste^11^ and maize silk powder^15^.

### Thermodynamic parameters

Standard enthalpy change (ΔH^0^) and standard entropy change (ΔS^0^) were calculated from the slope and the intercept of ln K_L_ versus 1/T plot presented in Supplementary Information, Figure S9. The values of these parameters and the standard Gibbs free energy change (ΔG^0^) are presented in Table 2. Negative values of ΔG^0^ indicate that the adsorption of methylene blue is a spontaneous and favorable process. The positive value for ΔH^0^ shows that the process is endothermic. The affinity of the adsorbent material for dye is reflected by the positive value of ΔS^0^ which indicates the increased randomness (the degrees of freedom of the adsorbed species) at the solid-solute interface. Similar result was reported in other previous studies^11,21,23,55–57^. The value of ΔH^0^ lower that 40 (kJ mol^−1^) indicate the physisorption is involved in methylene blue removal process from water^54,59^. Besides when ΔH^0^ < 20 (kJ mol^−1^) van der Waals interaction plays an important role in the physical adsorption process^60^.

**Table 2:** The values of the standard Gibbs free energy change (ΔG°), standard enthalpy change (ΔH°) and standard entropy change (ΔS°).

ΔG° (kJ mol^−1^)			ΔH° (kJ mol^−1^)	ΔS° (J mol^−1^)
285 K	296 K	306 K		
− 20.46	− 21.51	− 22.39	0.68	11.06

### Optimization parameters of adsorption process using Taguchi approach

**Figure 3:**
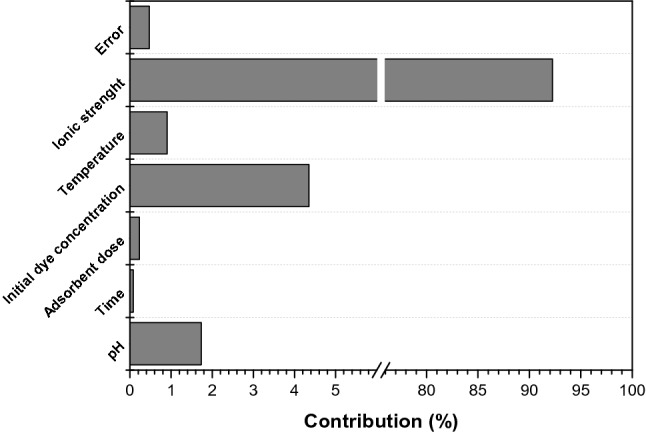
Contribution percentage of controllable factor influence on methylene blue removal process.

To perform the Taguchi method, 27 different experiments using L_27_ orthogonal array were run and the value for removal efficiency and S/N ratios of each run were determined (Supplementary Information, Table S2). The order of the controllable factors significance may be established by the rank of S/N ratio (Table 3). The most significant factor influencing the adsorption process was the ionic strength followed by initial dye concentration and pH. The optimum conditions to achieve the highest efficiency established using Taguchi method were: pH 10, contact time 10 (min), adsorbent dose 2.5 (mg g^−1^), initial dye concentration 50 (mg g^−1^), temperature 306 K and ionic strength 0.0 (mol L^−1^). The ANOVA results confirm the same order of controllable factor influence on methylene blue adsorption. The percentage contribution of each factor is given in Fig. 3.

**Table 3:** Response table for signal-to-noise S/N ratios (larger is better).

Level	pH	Time	Adsorbent dose	Initial dye concentration	Temperature	Ionic strength
1	33.30	33.93	33.63	34.81	33.42	38.13
2	33.88	33.72	33.92	33.64	33.95	33.19
3	34.39	33.91	34.01	33.12	34.19	30.24
Delta	1.09	0.21	0.38	1.69	0.77	7.90
Rank	3	6	5	2	4	1

### Desorption and regeneration study

The regeneration possibility of exhausted adsorbent was investigated using different desorbing agents (0.1 M HCl, distilled water and 0.1 M NaOH). HCl was a better desorbing agent for the regeneration of adsorbent (see Supplementary Information, Table S3). The regenerated adsorbent was tested for methylene blue adsorption (initial dye concentration: 100 mg L^−1^; pH: 7; contact time: 30 min; adsorbent dosage: 2 g L^−1^; temperature: 296 K), but process efficiency was reduced by 50%. Considering these results and the fact that Syringa *vulgaris* leaves powder is an inexpensive and easily available adsorbent it can be asserted that regeneration is not necessary.

## Conclusions

Adsorption capacity of *Syringa vulgaris* leaves powder is influenced by solution pH, contact time, initial dye concentration, adsorbent dose, ionic strength and is higher than other similar adsorbents. Langmuir isotherm and pseudo-second order kinetic model describe the adsorption process. Thermodynamic parameters indicate that adsorption is spontaneous and endothermic. Van der Waals interactions are implied in the physical adsorption process. The controllable factor with the most significant influence was ionic strength. Furthermore, the adsorbent is inexpensive and easily available, so it can be concluded that *Syringa vulgaris* leaves powder is an efficient low cost adsorbent for methylene blue removal from aqueous solutions.

## Supplementary information

Supplementary Information
