# Supplementary material for: Syringa vulgaris leaves powder a novel low-cost adsorbent for methylene blue removal: isotherms, kinetics, thermodynamic and optimization by Taguchi method
Source: Sci Rep. 2020 Oct 19;10:17676. doi: 10.1038/s41598-020-74819-x (PMC7810873; doi:10.1038/s41598-020-74819-x)
Supplement: Supplementary file 1 — Supplementary Information [file 41598_2020_74819_MOESM1_ESM.doc]

**Supplementary Information**

***Syringa vulgaris* leaves powder a novel low-cost adsorbent for methylene blue removal: isotherms, kinetics, thermodynamic and optimization by Taguchi method**

Giannin Mosoarca ^1^, Cosmin Vancea ^1^*, Simona Popa ^1^*, Marius Gheju ^1^, Sorina Boran ^1^

^1^ Politehnica University Timisoara, Faculty of Industrial Chemistry and Environmental Engineering, Bd. V. Parvan No. 6, 300223, Timisoara, Romania

^*^ Corresponding authors:

[cosmin.vancea@upt.ro](mailto:cosmin.vancea@upt.ro), Tel. +40256404194, Fax. +40 256 403 060;

[simona.popa@upt.ro](mailto:simona.popa@upt.ro), Tel. +40256404228, Fax. +40 256 403 060

**Table S1.** Controllable factors and their levels

| **Factor** | **Level 1** | **Level 2** | **Level 3** |
| --- | --- | --- | --- |
| pH | 2 | 6 | 10 |
| Time (min) | 10 | 30 | 50 |
| Adsorbent dose (mg L^-1^) | 0.5 | 1.5 | 2.5 |
| Initial dye concentration (mg L^-1^) | 50 | 150 | 250 |
| Temperature (K) | 285 | 296 | 306 |
| Ionic strength (mol L^-1^) | 0 | 0.1 | 0.2 |

**Table S2.** Experimental layout of L_27_ orthogonal array and results obtained for removal efficiency and S/N ratios

| **pH** | **Time** | **Adsorbent dose** | **Initial dye**  **concentration** | **Temperature** | **Ionic strength** | **Removal efficiency** | **S/N ratio** |
| --- | --- | --- | --- | --- | --- | --- | --- |
| 2 | 10 | 0.5 | 50 | 285 | 0 | 79.07 | 37.96 |
| 2 | 10 | 0.5 | 50 | 296 | 0.1 | 47.06 | 33.45 |
| 2 | 10 | 0.5 | 50 | 306 | 0.2 | 35.13 | 30.91 |
| 2 | 30 | 1.5 | 150 | 285 | 0 | 69.74 | 36.87 |
| 2 | 30 | 1.5 | 150 | 296 | 0.1 | 41.51 | 32.36 |
| 2 | 30 | 1.5 | 150 | 306 | 0.2 | 30.98 | 29.82 |
| 2 | 50 | 2.5 | 250 | 285 | 0 | 67.75 | 36.62 |
| 2 | 50 | 2.5 | 250 | 296 | 0.1 | 40.32 | 32.11 |
| 2 | 50 | 2.5 | 250 | 306 | 0.2 | 30.1 | 29.57 |
| 6 | 10 | 1.5 | 250 | 285 | 0.1 | 40.72 | 32.20 |
| 6 | 10 | 1.5 | 250 | 296 | 0.2 | 31.36 | 29.93 |
| 6 | 10 | 1.5 | 250 | 306 | 0 | 77.02 | 37.73 |
| 6 | 30 | 2.5 | 50 | 285 | 0.1 | 50.12 | 34.00 |
| 6 | 30 | 2.5 | 50 | 296 | 0.2 | 35.59 | 31.03 |
| 6 | 30 | 2.5 | 50 | 306 | 0 | 94.79 | 39.54 |
| 6 | 50 | 0.5 | 150 | 285 | 0.1 | 41.68 | 32.40 |
| 6 | 50 | 0.5 | 150 | 296 | 0.2 | 32.1 | 30.13 |
| 6 | 50 | 0.5 | 150 | 306 | 0 | 78.84 | 37.93 |
| 10 | 10 | 2.5 | 150 | 285 | 0.2 | 30.06 | 29.56 |
| 10 | 10 | 2.5 | 150 | 296 | 0 | 91.38 | 39.22 |
| 10 | 10 | 2.5 | 150 | 306 | 0.1 | 52.72 | 34.44 |
| 10 | 30 | 0.5 | 250 | 285 | 0.2 | 29.88 | 29.51 |
| 10 | 30 | 0.5 | 250 | 296 | 0 | 75.72 | 37.58 |
| 10 | 30 | 0.5 | 250 | 306 | 0.1 | 43.69 | 32.81 |
| 10 | 50 | 1.5 | 50 | 285 | 0.2 | 38.34 | 31.67 |
| 10 | 50 | 1.5 | 50 | 296 | 0 | 97.15 | 39.75 |
| 10 | 50 | 1.5 | 50 | 306 | 0.1 | 56.05 | 34.97 |

**Table S3.** The desorption efficiencies of methylene blue in different media

| **Desorption agent** | **Desorption efficiencies (%)** |
| --- | --- |
| HCl | 57.57 ± 1.51 |
| NaOH | 7.37 ± 0,17 |
| Distilled water | 1 ± 0,02 |

**Figure S1.** FT-IR spectrum of *Syringa vulgaris* leaves powder and methylene blue-loaded *Syringa vulgaris* leaves powder

**Figure S2.** Determination of point of zero charge (pHpzc) of *Syringa vulgaris* leaves powder

**Figure S3.** Langmuir and Freundlich isotherms (non-linear form) for methylene blue adsorption on *Syringa vulgaris* leaves powder (pH: 7; contact time: 30 min; adsorbent dosage: 2 g L^-1^;

temperature: 296 K)

**Figure S4.** Langmuir isotherm (linear form) for methylene blue adsorption on *Syringa vulgaris* leaves powder (pH: 7; contact time: 30 min; adsorbent dosage: 2 g L^-1^; temperature: 296 K)

**Figure S5.** Freundlich isotherms (linear form) for methylene blue adsorption on *Syringa vulgaris* leaves powder (pH: 7; contact time: 30 min; adsorbent dosage: 2 g L^-1^; temperature: 296 K)

**Figure S6.** Pseudo-first order and pseudo-second order kinetic models (non-linear form) for methylene blue adsorption on *Syringa vulgaris* leaves powder (initial methylene blue

concentration: 100 mg L^-1^, pH: 7; adsorbent dosage: 2 g L^-1^; temperature: 296 K)

**Figure S7.** Pseudo-first order kinetic models (linear form) for methylene blue adsorption on *Syringa vulgaris* leaves powder (initial methylene blue concentration: 100 mg L^-1^, pH: 7;

adsorbent dosage: 2 g L^-1^; temperature: 296 K)

**Figure S8.** Pseudo-second order kinetic models (linear form) for methylene blue adsorption on *Syringa vulgaris* leaves powder (initial methylene blue concentration: 100 mg L^-1^, pH: 7;

adsorbent dosage: 2 g L^-1^; temperature: 296 K)

**Figure S9.** Plot of ln K_L_ vs. 1/T for methylene blue adsorption on *Syringa vulgaris* leaves powder
